# Physiology, yield and nutritional contribution of hemp (*Cannabis sativa* L.) grown under different fertiliser types and environments

**DOI:** 10.1186/s42238-025-00273-z

**Published:** 2025-03-22

**Authors:** Mdungazi K. Maluleke, Kgaogelo R. Thobejane

**Affiliations:** College of Agriculture and Environmental Sciences, Department of Environmental Sciences, University of South Africa, Tshwane, 0002 South Africa

**Keywords:** Macronutrients, Recommended daily intake, Vitamins, Yield

## Abstract

The eradication of poverty and malnutrition are some of the main goals set by the United Nations through the Sustainable Development Goals (SDGs) 1 and 2. Humans have traditionally used *Cannabis sativa* L. for a variety of purposes, including medicine and as a raw ingredient for goods with added value such as drinks, cakes, and oil. The crop has gained considerable popularity in various industries due to its usage either as a fresh or processed material. The growing demand for *Cannabis sativa*’s raw materials for a range of applications has led to a steady increase in its cultivation. Because of this constant growing demand, it is essential that growers have a thorough awareness of all environmental conditions, particularly light intensity and the right fertiliser, for improvement of plant growth, yield and quality. Therefore, the study objective was to investigate the combined effect of different fertiliser types (chemical and organic) on the yield and biochemical constituents of *Cannabis sativa* under varying growing environments (shade net and open space), to enable comparative analysis to be done to assist growers in producing high-quality *Cannabis sativa* crops for commercial purposes. Fresh and freeze-dried samples were used to measure the yield and biochemical constituents. The treatment combination of shade net and chemical fertiliser resulted in superior inflorescence water content (40.2 g) and total phenols (14.7 GAE/100 g DW) compared to other treatments. Potassium content (989 mg/100 g DW) was superior under the treatment combination of chemical fertiliser and the open space environment compared to other treatments. Therefore, growers must consider the combination of light intensity and chemical fertiliser for yield and quality maximisation, whether under shade net or open space growing environments.

## Introduction

Light, soil nutrition, temperature and growing environments all have a direct impact on plant growth, physiological performance, yield and quality (Chandra et al. 2011; Eaves et al. 2020; Bevan et al. 2021; Danziger and Bernstein 2021a, b). In order to create an environment in which plants can maximise their physiological performance and improve their growth, yield and quality, growers should have a thorough understanding of how these aspects interact. The yield and quality of hemp (*Cannabis sativa* L.), a herbaceous plant belonging to the Cannabaceae family, depend on the interaction of factors such as the growing environment and fertiliser types (Kornpointner et al. 2021). The provision of essential macronutrients to plants, such as nitrogen (Saloner and Bernstein 2020, 2021), phosphorus (Shiponi and Bernstein 2021a, b), potassium (Saloner and Bernstein 2019; De Prato et al. 2022) and magnesium (Morad and Bernstein 2023), has a significant impact on the development and physiological function of cannabis plants during the vegetative and the reproductive growth stage. These macronutrients are also crucial for the crop’s production of secondary metabolites at the reproductive stage (Shiponi and Bernstein 2021b; Saloner and Bernstein 2022; De Prato et al. 2022). On the other hand, authors such as Vanhove et al. (2011), Maluleke (2022) and Wei et al. (2021) highlight climatic factors such as light and temperature as primary drivers affecting plant growth, development and quality. Since this plant is indigenous to Central Asia, but currently cultivated throughout the world due to its economic value, its adaptability to a combination of various conditions such as type of fertiliser and light intensity remains a subject of interest (Kepe 2003; Hourfane et al. 2023). The crop has gained a great deal of popularity in various industries due to its usage either as fresh or processed material (Moscariello et al. 2021; Arango et al. 2024). The multi-purpose utilisation of *Cannabis sativa* has played a pivotal role in human sustenance and economic growth globally (Kurnaz and Kurnaz 2021; Madden et al. 2022). This, according to Viviers et al. (2021), has led to a sharp increase in the production of the crop for different purposes such as consumption and processed value-added products such as drinks, oils and medicinal products. This addresses SDG 1 (no poverty) and SDG 2 (zero hunger), since fresh, raw and processed cannabis has been shown to contain a wealth of minerals, including calcium, iron, potassium, phosphorus, zinc, magnesium, ascorbic acid, beta-carotene, as well as vitamins A, E and D (Rupasinghe et al. 2020; Cerino et al. 2021; Krüger et al. 2022; Hourfane et al. 2023). The growing demand for the raw materials of *Cannabis sativa* for a range of applications has led to a steady increase in its cultivation (Campiglia et al. 2017). Because of this growing demand, producers are compelled to have a thorough awareness of all environmental conditions, particularly light intensity and the right fertiliser, to improve plant growth, yield and quality (Amaducci et al. 2008; Morello et al. 2022; Trancoso et al. 2022). Song et al. (2023) report that the chemical quality and production of cannabinoids and terpenes in *Cannabis sativa* are considerably affected by plant nutrition and the fertilisers applied during cultivation. Vanhove et al. (2011) and Magagnini et al. (2018) highlight light intensity as one of the factors contributing to the yield and quality of the crop. There are numerous reports that show the effect of individual factors such as light, irrigation, temperature and fertilisers on the growth, yield, and quality of *Cannabis sativa* (Morello et al. 2022; Rodriguez-Morrison et al. 2021). Deng et al. (2012) investigated the impact of a light intensity and fertiliser combination on sweet tea (*Cyclocarya paliurus*). They report significant variation in yield and quality, including biomass and flavonoids, when plants were exposed to different levels of light intensity and fertiliser. However, there is limited literature on the combined effects of growth environments and fertilisers on the physiology, yield, and quality of *Cannabis sativa* L. Therefore, the study objective was to investigate the combined effect of different fertiliser types on the physiology, yield, and biochemical constituents of *Cannabis sativa* grown under varying environments (shade net and open space), to allow comparative analysis to assist growers in producing high-quality *Cannabis sativa* crops for commercial purposes.

## Materials and methods

This study was carried out in 2023 in shade net and open space environments at the University of South Africa, Florida campus, Johannesburg, Gauteng, South Africa (lat. 26° 10’ 30” S, long. 27° 55’ 22.8” E). The minimum and maximum temperatures in the shade net (13–30 °C) and open space environments (14–34 °C) were recorded during the experimental period, shown in Table 1. Plants were exposed to natural light for a minimum of 10 to 12 h every day during the trial period (Table 1). The experiment was thus a complete randomised design with two factors: (i) different fertilisers (organic and inorganic/chemical) under varying growing environments (shade net and open space). Notably, plants cultivated on loam soil without fertiliser supply were used as the control treatment (negative control). Certified seeds obtained from the research institution (Cape Town, South Africa) were used to germinate seedlings for the experiment. Healthy, uniform seedlings of *Cannabis sativa*, which were 30 days old and germinated from peat substrate, were transplanted to pots 30 cm deep by 30 cm wide filled with loam soil under differing environments (shade net and open space) during spring to summer of 2023. Ten (10) replicates consisting of one plant per pot were used per treatment, resulting in 60 plants per growing environment. After the establishment of seedlings, plants were supplied with 5 g of granule fertilisers of different types, namely (i) organic fertiliser (GuanoBoost, Pretoria, South Africa) with nitrogen (N), phosphorus (P) and potassium (K) [N-3, P-2, K-5] and inorganic/chemical fertiliser (Nitrogreen KAN/LAN, Protek, Pretoria, South Africa) with nitrogen (N), phosphorus (P) and potassium (K) [N-2, P-3, K-2] (Table 1). Plants grown on loam soil with no fertiliser treatment were classified as negative control. The treatments were imposed two weeks later, after establishment. A calibrated container was used to manually irrigate the plant pots to ensure that water was distributed evenly during the experiment. The pot size/dimension is illustrated in the formula below:

**Table 1:** Meteorological data of shade net and open space environments

	Open space (L1)			
	T_max_ (˚C)	T_min_	Rainfall (mm)	Light intensity
**Month**				
September	30 ˚C	14 ˚C	27.2	823
October	31 ˚C	16 ˚C	97.1	477
November	32 ˚C	19 ˚C	79.3	599
December	33 ˚C	20 ˚C	140.3	1 156
			**516.4**	**3 055**
	**Shade net (L2)**			
September	28 ˚C	13 ˚C	10	411
October	29 ˚C	15 ˚C	22	245
November	27 ˚C	15 ˚C	68	210
December	30 ˚C	16 ˚C	119	270
			**337**	**1 136**

Tmax = maximum temperature. Tmin = minimum temperature. °C = degree Celsius

1 $$\begin{aligned}&{\rm{Area}}\left( {{\rm{depth }} \times {\rm{ width}}} \right)\,{\rm{30 }}\,{\rm{cm}}\, \times \,{\rm{30\ cm}}\,{\rm{ = }} {\rm{900}}\,{\rm{c}}{{\rm{m}}^{\rm{2}}}{\rm{,}}\,\cr&\quad{\rm{A}}\,{\rm{ = \rm pi\:\left(\frac{d}{2}\right)\times\:2}}\,{\rm{d}}\,{\rm{ = }}\,{\rm{286}}{\rm{.5}}\,{\rm{c}}{{\rm{m}}^{\rm{2}}}\,{\rm{planting}}\,{\rm{pots}}\end{aligned}$$

Soil mineral analysis (Table 1) was carried out at the Agricultural Research Council (25° 44’ 19.4” S 28° 12’ 26.4” E), Arcadia, Pretoria.

### Physiological parameters

#### Chlorophyll content

During the experiment, chlorophyll content was measured once during the inflorescence stage. The amount of chlorophyll on three leaves per plant was measured in the morning on day 5 of every week using a leaf chlorophyll meter (CCM-200 plus Opti-Sciences, Inc., Hudson, NH, USA). Because the top leaf surface has a higher chlorophyll content than the lower leaf surface, the adaxial or upper-leaf surface was measured four times in triplicate, the average value was computed, and the results recorded as followed by Maluleke (2022) and Tuckeldoe et al. (2023).

#### Stomatal conductance

During the experiment, stomatal conductance was measured once during the inflorescence stage. According to Zhang et al. (2020) and Tuckeldoe et al. (2023), stomatal opening and conductance activities are more prominent on the abaxial, or lower-leaf surface, and therefore only this surface was evaluated, according to the method adopted by Tuckeldoe et al. (2023). Every week on the fifth day, in the morning, a porometer (Delta-T Device, AP4 Leaf Porometer, Cambridge, United Kingdom) was used to measure the stomatal conductance of three leaves per plant.

#### Plant height

The *Cannabis sativa* plant height in centimetres (cm) was measured once a week in the morning using a measuring tape (Stanley Fatmax, Claremont Cape Town, South Africa).

#### Stem diameter

Using a vernier calliper (Digimatic 150 mm, Epacon Supplies Pty, Edenvale, South Africa), the stem diameter in millimetres (mm) of the plants undergoing treatment was continually recorded every fifth day of the week. In short, the calliper was set up on aluminium and “invar” (a nickel and iron alloy with low thermal expansion) holders. Elastic straps were utilised to secure the holders to the plant stems, and a sealing paste was applied to the plant stem surface to connect the sensor needle to it. This was done following the procedure of Yang et al. (2021) with minor modification (triplicates).

### Yield components

#### Total biomass and water content

Using an electronic scale (Uni-Bioc, China), the fresh biomass of inflorescence in grams was weighed at the end of the experiment. After initial weighing, the inflorescence was then transferred to the cud box covered with foil and placed for 72 h in an 80 °C oven. Following the procedure of Tuckeldoe et al. (2023), the total biomass was determined by adding the above-ground (dry) biomass with inflorescence (dry) biomass (Eq. 2). For water content determination, the *Cannabis sativa* inflorescence (fresh) biomass was subtracted from inflorescence (dry) biomass using the procedure of Tuckeldoe et al. (2023) as shown in Eq. 3.

2 $$\eqalign{\rm {Total}\,\rm {biomass} &= \,\rm {Above} - \rm {ground}\,biomass\,\left( {dry} \right)\, \cr & + \,\rm {Inflorescence}\, \rm {biomass}\,\left( \rm {dry} \right) \cr} $$

3 $$\eqalign{\rm {Water}\,\rm {content} &= \,\rm {Fresh}\,\rm {inflorescence}\,\rm {biomass} \cr & - \rm {Dry}\,\rm {inflorescence}\,\rm {biomass} \cr} $$

#### Harvest index

The harvest index of *Cannabis sativa* was determined by using the procedure of Tuckeldoe et al. (2023). Briefly, *Cannabis sativa* inflorescence (dry) biomass was divided by total biomass as shown in Eq. 4 below:

4 $${\rm{HI = }}{{{\rm {Inflorescence\,\rm{dry}\,\rm{ biomass}}}\left( {{\rm {dry}}} \right)} \over {{\rm {Totalbiomass}}}}$$

### Biochemical constituents and nutritional analysis

Biochemical constituents and nutritional analysis of *Cannabis sativa* L. harvested from varying fertiliser types under different growing environments was carried out in January 2022 using freeze-dried samples. *Cannabis sativa* L. inflorescence was used for analyses of vitamin C, vitamin A, total flavonoids, total phenols, macro- and micro-nutrients. For vitamin C, which was expressed in milligram per 100 g (mg/100 g) of dry weight (DW), the method followed by Martin et al. (2000) and Tuckeldoe et al. (2023), with slight modification (triplicates), was used. Regarding vitamin C, expressed in milligram per 100 g of dry weight, the procedure followed by Martin et al. (2000), adopted by Tuckeldoe et al. (2023), with slight modification (triplicates), was followed. Regarding total flavonoids, expressed in mg catechin equivalents (CE) per dry weight (DW), the method followed by Maluleke et al. (2021), adopted by Tuckeldoe et al. (2023), was used with slight modification (triplicates). For determination of total phenolic content, expressed in mg of garlic acid equivalents (GAE) per grams of dry weight (DW), the method followed by Maluleke et al. (2021), adopted by Tuckeldoe et al. (2023), was utilised with minor modification (triplicates). Regarding the macro- and micro-nutrients content, expressed in mg/100 g DW, the method followed by Moyo et al. (2018), adopted by Maluleke et al. (2021) and Tuckeldoe et al. (2023), was utilised with minor modification (triplicates).

### Statistical analysis

A two-way analysis of variance (ANOVA) was used to analyse the growth and yield of *Cannabis sativa L.* grown in two different fertilisers (organic and inorganic/chemical fertilisers) under varying growing environments (shade net and open space). Generalised linear mixed-model procedures for GenStat (version 22.1, 2023, VSNI, UK) were used for data analysis. The model was used to assess the fixed effects of fertiliser types and the growing environment on the studied variables (chlorophyll content, stomatal conductance, total biomass, harvest index, plant height, stem diameter and inflorescence water content). The Shapiro-Wilk and Bartlett tests were used to check the normality and homogeneity of variance. A two-way ANOVA was used to analyse data on the biochemical constituents and nutritional content of the *Cannabis sativa* inflorescence harvested from varying fertiliser types (organic and inorganic/chemical) under different growing environments (shade net and open space). The variables measured included vitamin C, vitamin E, total flavonoids, total phenols and macro- and micro-nutrients. The least significant difference (LSD) was considered for all studied variables. StatSoft (USA) version 10 was utilised for all statistical analysis.

## Results and discussion

### Physiological components

#### Chlorophyll and stomatal conductance

**Fig. 1:**
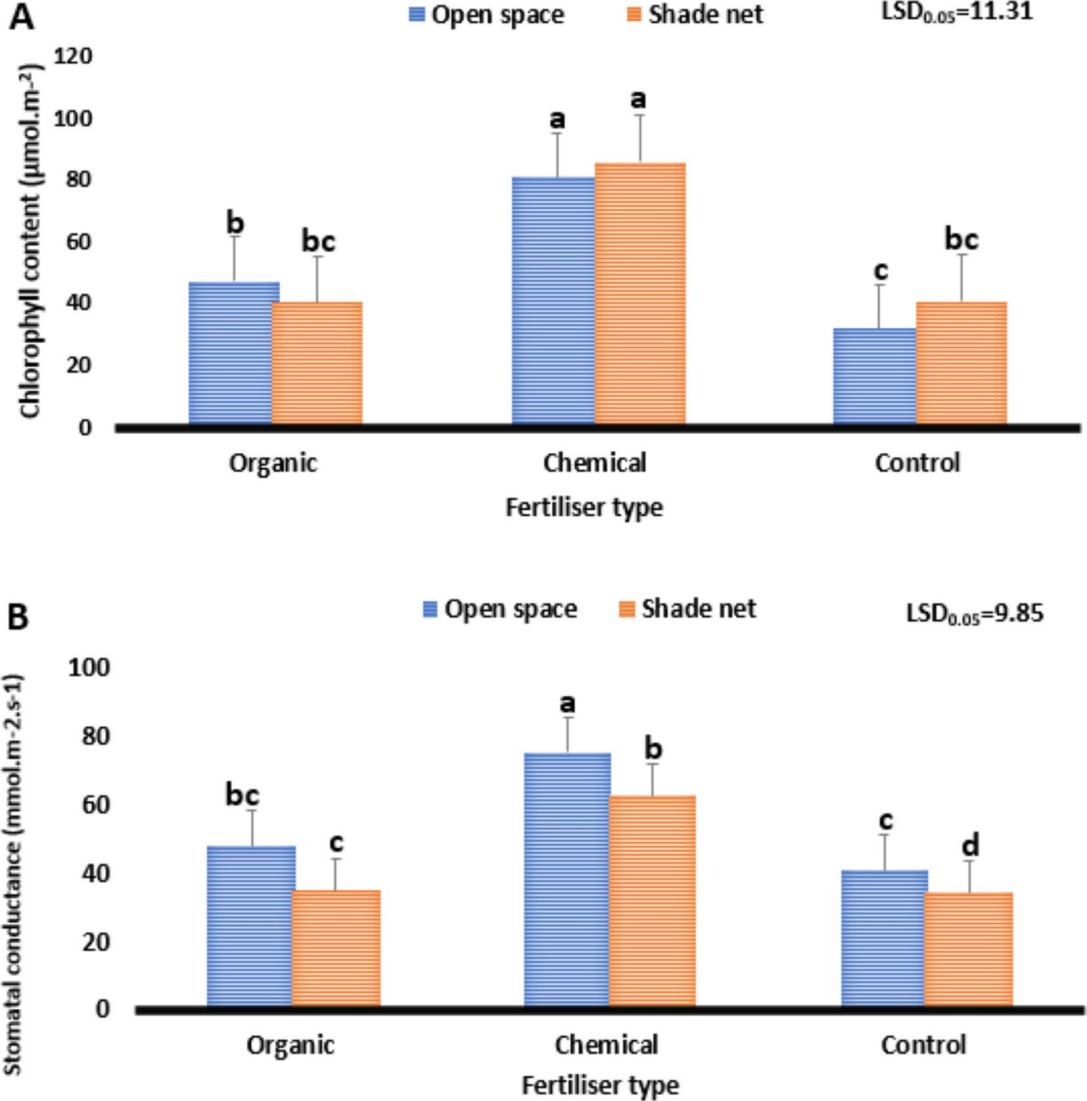
Effect of varying fertiliser types on the physiological performance of *Cannabis sativa*. Chlorophyll content (**A**). Stomatal conductance (**B**). The least significant difference, or LSD0.05, is used. Means with different letters (a, b, c) are significantly different at *P* ≤ 0.05

Figure 1 shows the impact of various fertiliser types on the chlorophyll content and stomatal conductance of *Cannabis sativa* L. grown in different growing environments. The difference in chlorophyll content and stomatal conductance was found to be significant (*P* ≤ 0.05). Regarding chlorophyll content, results ranged from 31.8 to 85.7 µmol.m^-2^. Furthermore, those plants grown on loam soil (control) under the open space environment reduced chlorophyll content from 85.7 to 31.8 µmol.m^-2^, whereas the treatment combination of the shade net environment and chemical fertiliser increased it from 31.8 to 85.7 µmol.m^-2^.

The variation between the highest (85.7) and lowest (31.8) chlorophyll content was 53.9 µmol.m^-2^. The chloroplast thylakoids, which house the chlorophyll pigments, are where the light reactions in plants take place (Tuckeldoe et al. 2023). The pigment molecules’ electrons become energetic when light energy enters them, and these electrons are then transferred through the electron transport chain located in the thylakoid membrane (Maluleke 2022). Plants constantly manufacture chlorophyll to maintain the net photosynthesis rate in their leaves (Shu et al. 2013). A contributing element to the change in chlorophyll content was found to be temperature and light intensity. The open space environment had a maximum temperature of 32 °C, and the shade net environment was 28.6 °C, which varied by 3.4 °C (Table 1). Regarding light intensity, the shade net environment light intensity (1 460) and open-space environment (4 202) varied by 2 742 lx (Table 1). These findings align with those of Ping et al. (2015) and Maluleke (2022), who observed fluctuations in chlorophyll concentration of apple and cucumber plants grown under diverse environmental circumstances with varied light intensity.

For stomatal conductance, results ranged from 34.5 to 75.3 mmol.m^-2^.s^-1^. In addition, plants grown on loam soil (control) under the shade net environment had the lowest conductance (34.5 mmol.m^-2^.s^-1^), whereas the treatment combination of chemical fertiliser and the open space environment demonstrated the highest conductivity of 75.3 mmol.m^-2^.s^-1^, compared to other treatments. The variation between the highest (75.3) and lowest (34.5) stomatal conductance was 40.8 mmol.m^-2^.s^-1^. As guard cells expand due to water absorption, stomatal pores typically open and close when guard cells contract (Maluleke et al. 2021). Light intensity, temperature, humidity and carbon dioxide levels are a few variables that affect stomatal opening and closing, according to Shu et al. (2013). With adjustments to the stomatal pore size, stomata govern both the exchange of gases between the plant and its surroundings as well as the loss of water, subsequently affecting the net photosynthesis rate, causing an increase or decrease in the plant’s overall yield (Tuckeldoe et al. 2023). The open space environment’s light intensity (4 202 lx), which was 2 742 higher than that of the shade net environment, proved to be the main contributing factor to the variation in the stomatal conductance performance among plants (Table 2). These observations are in line with those of Fu et al. (2017) and Maluleke (2022), who report a variation in stomatal conductance of cucumber and lettuce plants subjected to different light intensities and environmental conditions.

**Table 2:** Mineral analysis (mg.kg^-1^) of experimental soil for *Cannabis sativa* experiment

	Mineral composition analysis (mg.kg^-1^)							
Fe	Mn	Cu	Zn	P	Ca	Mg	K	Na
38	17	14	15	25	1 528	223	223	58

mg = milligrams. kg = kilograms

#### Plant height and stem diameter

**Fig. 2:**
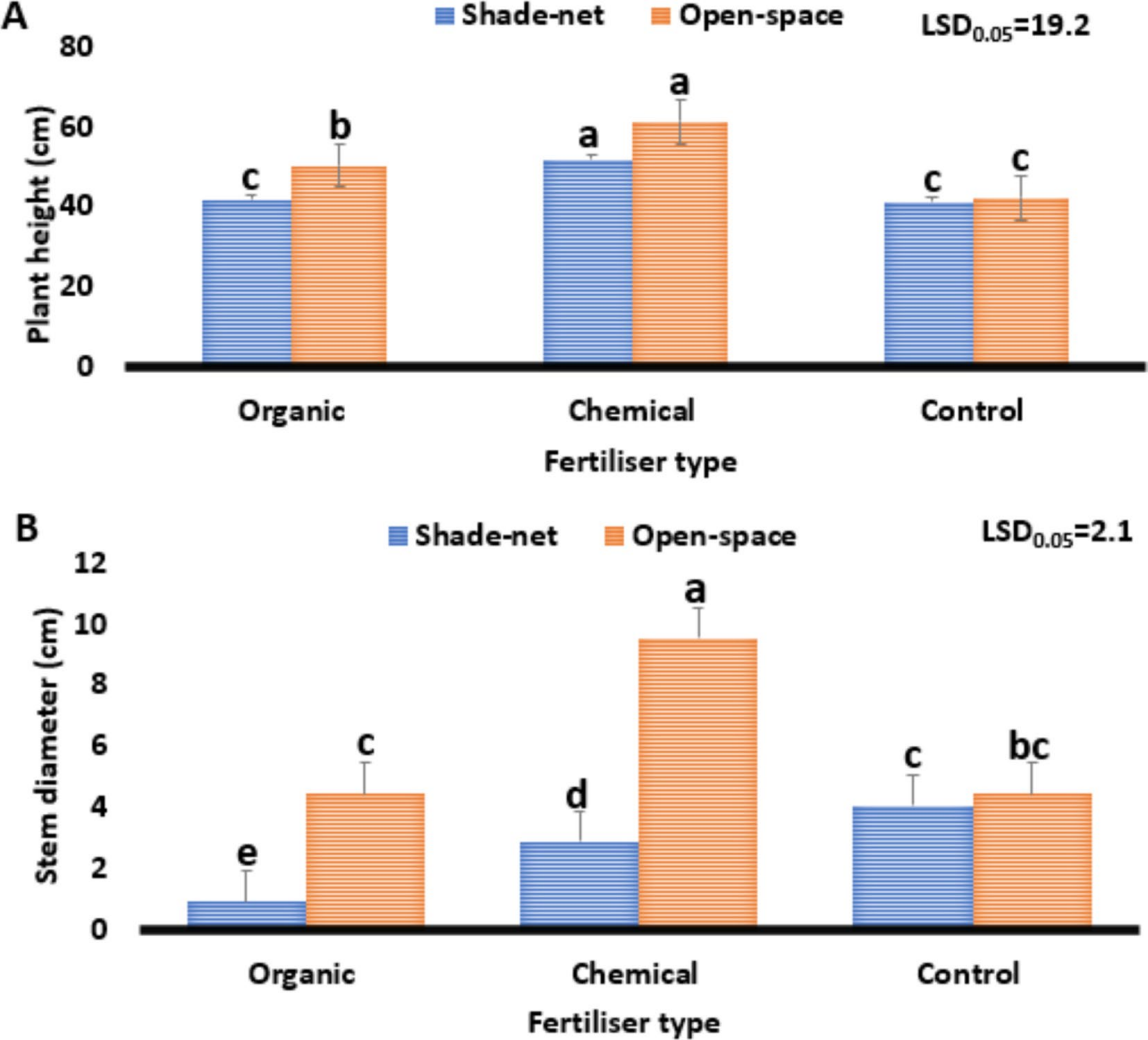
Effect of varying fertiliser types on the growth performance of *Cannabis sativa*. Plant height (**A**). Stem diameter (**B**). The least significant difference, or LSD0.05, is used. Means with different letters (a, b, c) are significantly different at *P* ≤ 0.05

The effect of the different fertiliser types on the plant height and stem diameter of *Cannabis sativa* grown under varying environments is shown in Fig. 2 (A and B). Study results show significant differences (*P* ≤ 0.05) in the effect of different fertiliser types on the plant height and stem diameter of *Cannabis sativa* grown under different environments. In terms of plant height (Fig. 2A), results ranged from 41.2 to 61.3 cm. Additionally, plants grown under loam soil (control) had reduced plant height from 61.3 to 41.2 cm, whereas the treatment combination of chemical fertiliser and the open space environment increased it from 41.2 to 61.3 cm. Plant growth and development are significantly influenced by light (Reichel et al. 2021). It is critical to many processes, including photosynthesis, which yields carbohydrates, a substance that plants need for respiration. It is also critical to several other processes, including plant growth, development and hormone distribution control (Tang et al. 2017). According to the study findings, there was a 3.4 °C difference in temperature between the open space (4 202) and the shade net (1 460) environments (Table 2), whereas there was only a 2 742 lx variation in light (Table 2). When compared to other treatments, the values derived from this study demonstrate that the combination of high light intensity, temperature, and phosphorus content of chemical/inorganic fertiliser under the open space environment was the primary contributor to plant height differences. These findings are in line with those of Trouwborst et al. (2010); Saloner and Bernstein (2020); Danziger and Bernstein 2021c); Paradiso and Proietti (2022); Song et al. (2023), who found variation in plant response in terms of height, stem diameter and leaf area of plants grown under varying photoperiod regimes.

On the other hand, there was a 3 °C difference in temperature between the open space environment (31.5) and the shade net environment (28.5), and there was only a 2 742 lx variation in light (Table 1). When compared to other treatments, the values derived from this study demonstrate that the combination of high light intensity, temperature, and phosphorus content of chemical/inorganic fertiliser under the open space environment was the primary contributor to plant height differences. These findings support those of Inthichack et al. (2013); Saloner and Bernstein (2019); Danziger and Bernstein (2021a), who found variation in plant response regarding growth such as plant height of cannabis, eggplant, sweet pepper and tomato subjected to varying photoperiod regimes. 

Regarding stem diameter (Fig. 2B), study results ranged from 0.95 to 9.55 cm. Furthermore, the treatment combination of organic fertiliser and the shade net environment reduced the stem diameter from 9.55 to 0.95 cm. The thickest stem diameter was found in the treatment combination of chemical fertiliser and the open space environment. The variation between the lowest (0.95) and highest (9.55) stem diameter was 8.6 mm. All plant cells contain the nutrient phosphorus, which is essential for development and growth (Moher et al. 2022). Additionally, it plays a crucial role in several essential plant processes, such as the flow of nutrients inside plant cells, energy transfer, photosynthesis and sugar transformation (Ahmad et al. 2015). The variation between the lowest (41.2) and highest (61.3) plant height was 20.1 cm, and the phosphorus content variation among fertilisers (organic 4%) and (inorganic/chemical 7.3%) was 3.3%. The values obtained from this study suggest that the mineral phosphorus from chemical/inorganic fertiliser, combined with higher light intensity (4202) from the open space environment, was the contributing factor for the variation in stem diameter of *Cannabis sativa*, when compared to other treatments. Moreover, study findings indicate that high phosphorus content from chemical fertiliser enabled the plant to easily facilitate transportation of resources such as water, air and nutrient movement, which move primarily from the roots to the stems and leaves. These findings support those of Vanhove et al. (2011), Fu et al. (2017), Danziger and Bernstein (2021a) and Kakabouki et al. (2021), who found variation in height and shoots of *Cannabis sativa* and lettuce plants grown in differing light intensity and fertiliser concentrations.

### Yield components

#### Total biomass, harvest index and water content

**Fig. 3:**
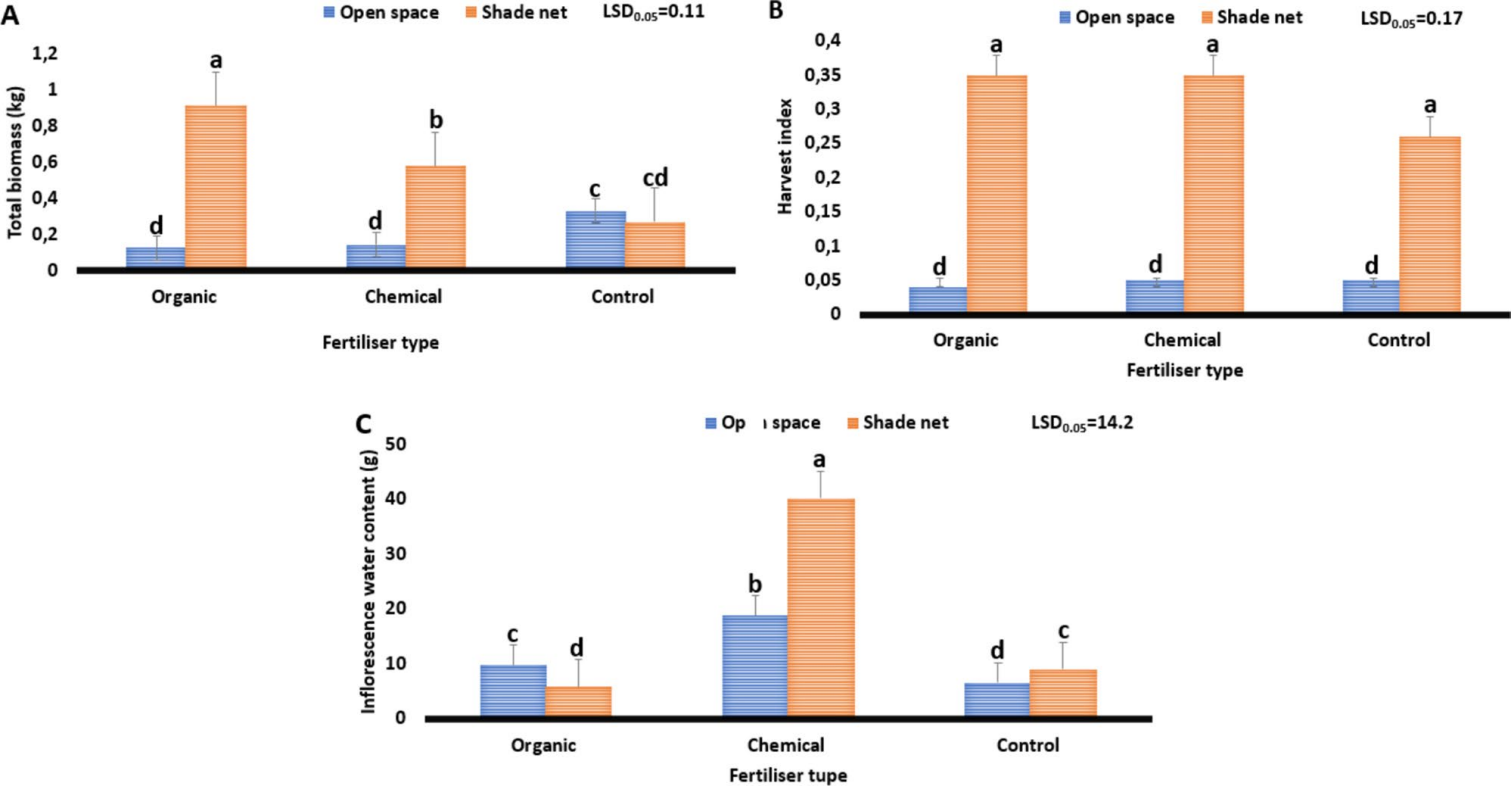
Effect of varying fertiliser types on the yield components of *Cannabis sativa* grown under different environments. Total biomass (**A**). Harvest index (**B**). Inflorescence water content (**C**). The least significant difference, or LSD0.05, is used. Means with different letters (a, b, c) are significantly different at *P* ≤ 0.05

Figure 3 illustrates the effect of different fertiliser types on the yield components (total biomass, harvest index and water content) of *C. sativa* grown in various environments. The results show a significant difference (*P* ≤ 0.05) amongst treatments on the total biomass, harvest index and water content of *C. sativa* grown under varying environments. Study results show that total biomass ranged from 0.9 to 0.58 kg and a treatment combination of organic fertilisers and shade net reduced the total biomass from 0.58 to 0.9 kg. There was a noticeable increase under the treatment combination of chemical fertiliser and open space environment, which increased the total biomass from 0.9 to 0.58 kg. It has been established that minerals, such as phosphorus, have a direct impact on the total biomass of plants (Kakabouki et al. 2021). Based on the study findings, the phosphorus content ratio amongst fertiliser types (organic − 4% and inorganic/chemical − 7.3%), which varied by 3.3%, proved to be a contributing factor to total biomass variation of *C. sativa* grown under varying environments (Table 3). These findings are in line with those of Forrest and Young (2008); Bernstein et al. (2019); Danziger and Bernstein (2021a), who found variation in plant biomass of plants grown under different fertiliser types and ratios.

**Table 3:** Different fertiliser types used for the experimental treatment

Organic fertiliser	Ratio	NPK content per treatment	Total application of elements (mg/g of soil)
Nitrogen (N) = 3	6	7.2	1.44
Phosphorus (P) = 2	4	4.8	0.96
Potassium (K) = 5	10	12	2.4
**Total = 20%**			
**Chemical fertiliser**	**Ratio**	**Mineral concentration**	**Total application of elements (mg/g of soil)**
Nitrogen (N) = 2	4.9	5.8	1.16
Phosphorus (P) = 3	7.3	8.8	1.76
Potassium (K) = 2	4.9	5.8	1.76
**Total = 17%**			

N = nitrogen. P = phosphorus. K = potassium. mg = milligram. g = gram

For harvest index, results ranged from 0.04 to 0.57. Furthermore, the treatment combination of the open space environment combined with organic fertiliser reduced the harvest index from 0.57 to 0.04, whereas the treatment combination of shade net environment combined with chemical fertiliser increased it from 0.04 to 0.57. The variation between the lowest (0.04) and highest (0.57) harvest index was 0.53. The superior harvest index proved to have been caused by varying potassium content from fertilisers (organic − 10% and inorganic/chemical − 4.9%), which varied by 5.1% (Table 3), with shade net proving to be the favourable environment. Potassium is a macronutrient responsible for (i) better water and nutrient movement within the plant, (ii) an increase of carbohydrates in plant tissue and (iii) better production of ATP, which regulates the rate of photosynthesis (Maluleke 2022). These findings align with those of Ramadan et al. (2014) and Lustosa Filho et al. (2020), who report a variation in the harvest index of plants grown under varying fertilisers and environmental factors.

Regarding water content, the study results ranged from 5.8 to 40.2 g. Moreover, the treatment combination of the shade net environment and organic fertilisers resulted in lower water content (5.8 g), whereas the treatment combination of the shade net environment and chemical fertiliser obtained the highest water content (40.2 g), compared to other treatments. The variation between the lowest (5.8) and highest (40.2) harvest index was 34.4 g. It has been established that crop water availability and fertilisers, especially potassium, the main nutrient that helps the plant control biomass accumulation, enhances inflorescence size (Tuckeldoe et al. 2023). The variation in inflorescence water content proved to have been caused by varying potassium content ratios within fertilisers (organic − 10% and inorganic/chemical − 4.9%), which varied by 5.1% as shown in Table 3. These findings align with those of El-Mageed et al. (2015, who discovered that plant competition for resources like light and water leads to variations in fruit weight and size.

### Biochemical constituents of cannabis sativa inflorescence and its potential contribution to human nutrition

#### Vitamin C, vitamin E, total flavonoids and total phenols

**Fig. 4:**
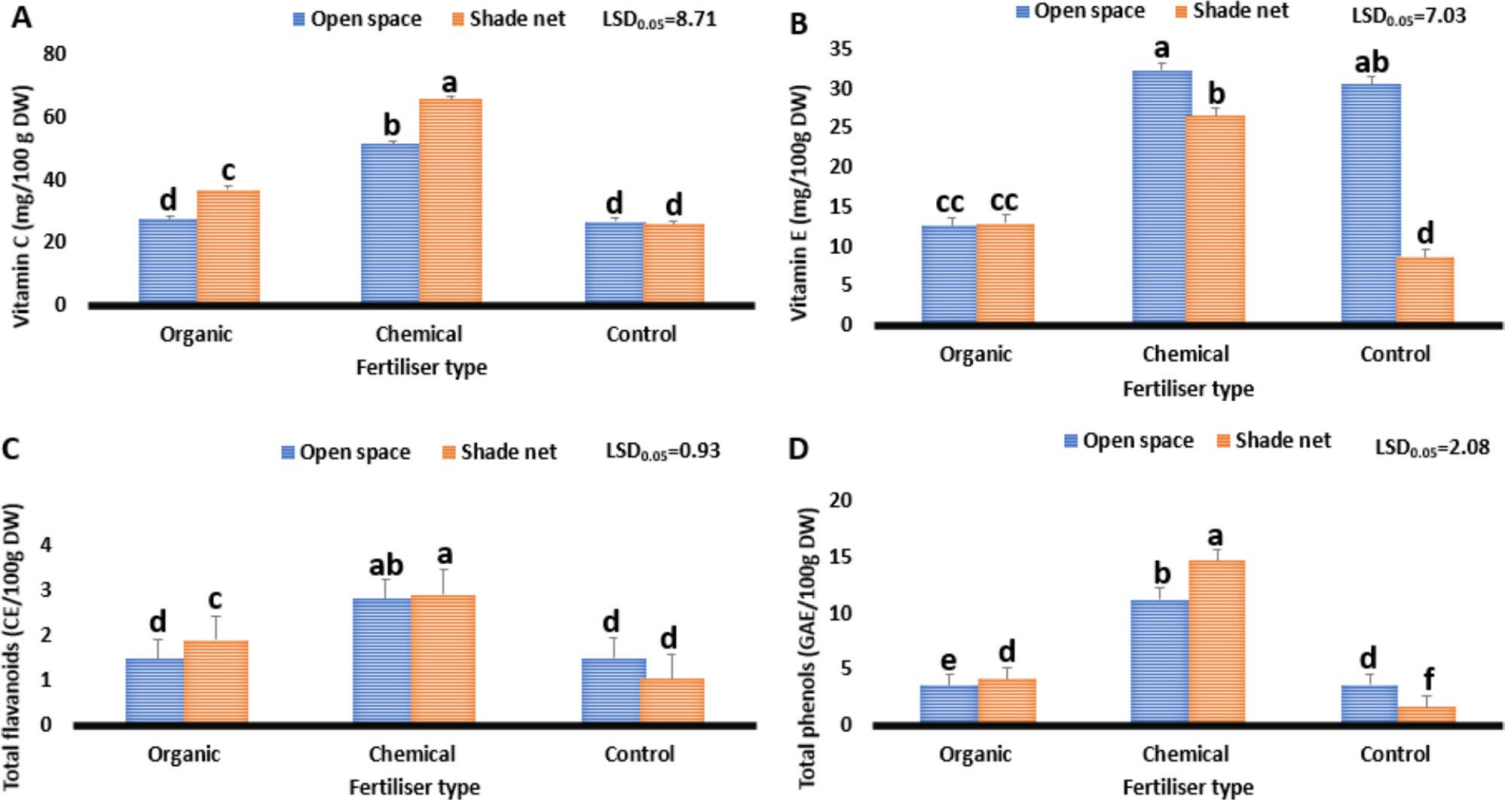
Effect of varying fertiliser types on the biochemical constituents of *Cannabis sativa* grown under different environments. Vitamin C (**A**). Vitamin E (**B**). Total flavonoids (**C**). Total phenols (**D**). The least significant difference or LSD0.05, is used. Means with different letters (a, b, c) are significantly different at *P* ≤ 0.05

Figure 4 presents the effect of different fertiliser types on the biochemical constituents (vitamin C, vitamin E, total flavonoids, and total phenols) on *C. sativa* grown under varying environments. Study results indicate significant variation (*P* ≤ 0.05) in biochemical constituents among treatments. In terms of vitamin C, results ranged from 26 to 66 mg/100 g DW. Additionally, loamy soil (control) and the shade net environment reduced vitamin C from 66 to 26 mg/100 g DW, whereas the treatment combination of the shade net environment and chemical fertiliser increased it from 26 to 66 mg/100 g DW. The variation between the highest vitamin C content (66) and recommended daily intake (95) was 29 mg. Values obtained from this study suggest that *C. sativa* could contribute approximately 69% of the vitamin C required by humans daily. Every tissue in the human body requires vitamin C to develop and repair (Tuckeldoe et al. 2023). In addition, it is utilised in the formation of collagen, a crucial protein that forms blood vessels, tendons, ligaments, and skin (Maluleke et al. 2024). Furthermore, it is essential for the formation of scar tissue and the healing of wounds (Uusiku et al. 2010). Findings from this study indicate that consumption of *Cannabis sativa* and value-added products grown in a shade net environment with chemical fertilisers could potentially assist in curbing conditions such as skin challenges, excessive bruising, poor wound healing, and swollen joints, which are symptoms linked to inadequate vitamin C in the human diet (Achaglinkame et al. 2019; Tuckeldoe et al. 2023; Maluleke et al. 2024).

Regarding vitamin E, study results ranged from 8.7 to 32 mg/100 g DW. Moreover, loam soil (control) combined with the shade net environment reduced vitamin E from 32.3 to 8.7 mg/100 g DW, whereas the treatment combination of an open space environment and chemical fertiliser increased it from 8.7 to 32.3 mg/100 g DW. The variation between the highest vitamin E (32.3) and the recommended daily intake (22) was 10.3 mg. Findings obtained from this study suggest that *Cannabis sativa* contains 10.3 mg more vitamin E than the recommended daily intake. Due to its antioxidant properties, anti-inflammatory properties and capacity to boost human immunological function, vitamin E has been shown to be highly useful in the prevention and reversal of a number of diseases in the human body (Achaglinkame et al. 2019; Tuckeldoe et al. 2023). Therefore, consumption of *Cannabis sativa* grown in an open space environment with organic fertiliser could assist in curbing conditions such as muscle pain and constant fatigue, which are conditions linked to low vitamin E in the human diet (Goulas and Manganaris 2012).

For total flavonoids, study results ranged from 1.02 to 2.9 CE/100 g DW. Loam soil (control) and the shade net environment reduced total flavonoids from 2.9 to 1.02 CE/100 g DW, whereas the treatment combination of the shade net environment and chemical fertiliser increased it from 1.02 to 2.9 CE/100 g DW. The difference between the highest total flavonoids (2.9) and the recommended daily intake (225) was 252.5 mg. This means that *Cannabis sativa* grown under the treatment combination of the shade net environment and chemical fertiliser, which obtained the highest concentration, could contribute about 1.3% of the total flavonoids required in the daily human diet. Biological substances called flavonoids provide several health advantages, such as antiviral, anticancer and antioxidant qualities (Maluleke et al. 2024). Additionally, they also have cardio- and neuroprotective properties which aid in the enhancement of human health (Tuckeldoe et al. 2023). Even though values obtained from this study were lower compared to the recommended daily intake, consumption of *Cannabis sativa* and value-added products grown in the shade net environment with chemical fertiliser could assist in curbing conditions such as cardiovascular disease and diabetes, which are linked to low flavonoids in the human diet (Maluleke et al. 2021).

Concerning total phenols, study results ranged from 1.6 to 14.7 GAE/100 g DW. In addition, loam soil (control) combined with the shade net environment reduced total phenols from 14.7 to 1.6 GAE/100 g DW. The highest total phenolic content (14.7 GAE/100 g DW) was observed under the treatment combination of the shade net environment and chemical fertiliser. The variation between the highest total phenolic content (14.7) and the recommended daily intake (1000) was 985.3 mg. Phenolic compounds are essential for defensive mechanisms including anti-ageing, anti-inflammatory, antioxidant and antiproliferative actions from a physiological perspective (Smeriglio et al. 2016; Al Khoury et al. 2021). The values obtained from this study mean that *Cannabis sativa* grown in the shade net environment with organic fertiliser could contribute about 1.5% of the recommended phenols required by humans daily. Even though the values obtained are lower, consumption of *Cannabis sativa* value-added products could potentially assist in curbing kidney damage and skin burns, which are linked to low phenols in the human diet.

### Macro- and micro-nutrients

#### Calcium, magnesium, phosphorus, potassium and iron

**Fig. 5:**
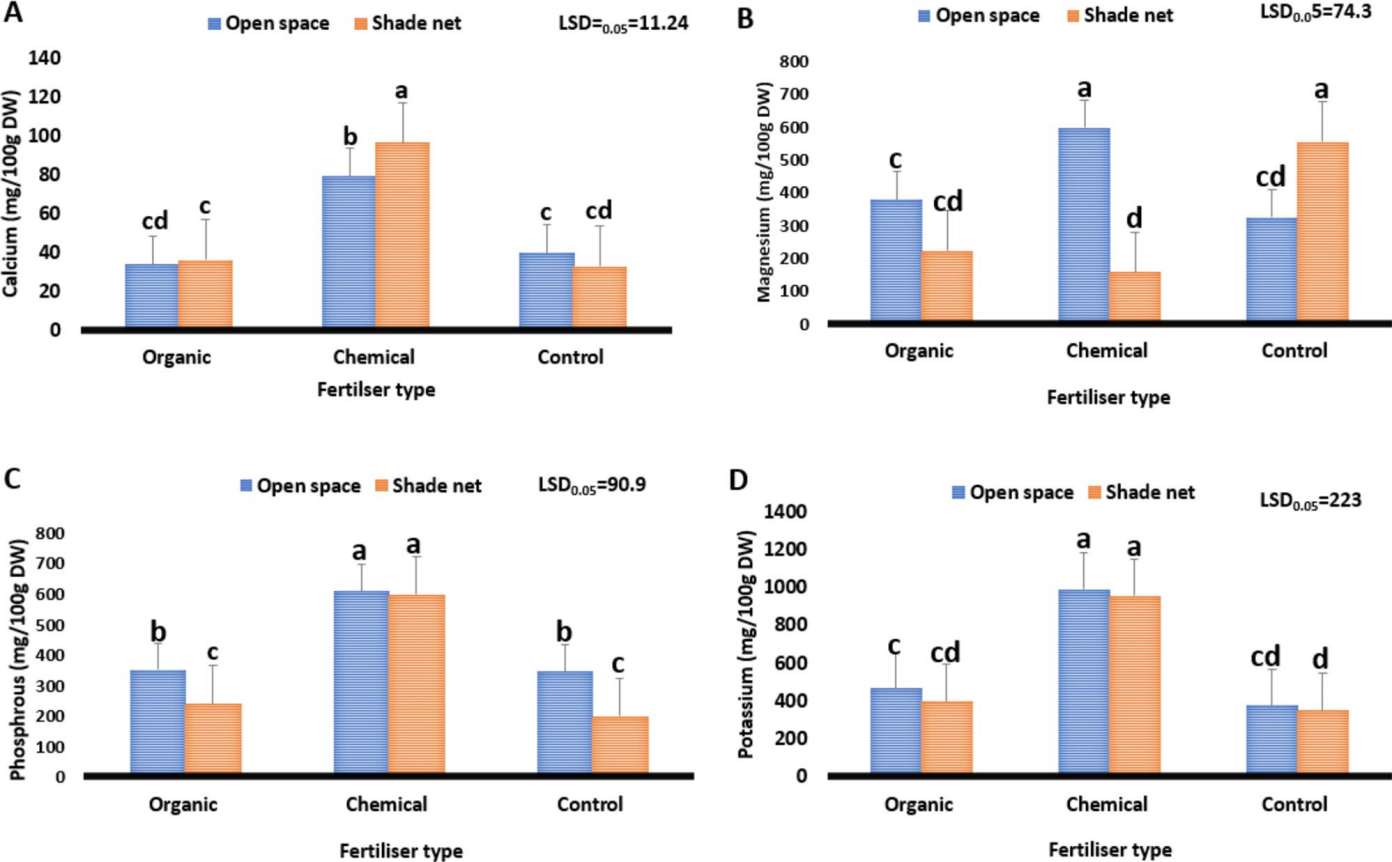
Effect of varying fertiliser types on the macro-nutrients (mg/100 DW) of *Cannabis sativa* grown under different environments. Calcium (**A**). Magnesium (**B**). Phosphorus (**C**). Potassium (**D**). The least significant difference, or LSD0.05, is used. Means with different letters (a, b, c) are significantly different at *P* ≤ 0.05

The effect of the different fertiliser types as well as the growing environments on the macro- and micro-nutrients of *Cannabis sativa* is shown in Fig. 5. Study results show significant differences (*P* ≤ 0.05) in the macro- and micro-nutrients (calcium, magnesium, phosphorus, potassium, and iron) of *Cannabis sativa* grown with varying fertiliser types under different growing environments. For calcium, study results ranged from 32.8 to 96.7 mg/100 g DW. Loam soil combined with the shade net environment reduced calcium content from 96.7 to 32.8 mg/100 g DW, whereas the treatment combination of chemical fertiliser and the shade net environment increased it from 32.8 to 96.7 mg/100 g DW. Calcium is required for the human body to contract its muscles and for its nerves to send information from the brain to every part of the body (Maluleke et al. 2024). Furthermore, calcium facilitates the release of hormones that affect a wide range of physiological processes and supports the blood vessels that transport blood throughout the body (Tuckeldoe et al. 2023). The variation between the highest calcium content (96.7) and the recommended daily intake (1 150) was 1 053 mg. The values obtained from this study mean that *Cannabis sativa* inflorescence could contribute about 8.3% of the calcium required by humans daily. Furthermore, although the calcium content of *Cannabis sativa* was lower, consuming *Cannabis sativa* and its value-added products harvested from a shade net environment with chemical fertilisers may help reduce symptoms like muscle cramps, stomach problems, frequent urination, and appetite loss, which are associated with inadequate calcium intake in the human diet (Achaglinkame et al. 2019).

Regarding magnesium, study results ranged from 157 to 597 mg/100 g DW. Moreover, a treatment combination of the shade net environment and chemical fertiliser reduced magnesium content from 597 to 157 mg/100 g DW, whereas the open space environment combined with chemical fertiliser increased it from 157 to 597 mg/100 g DW. The variation between the highest magnesium content (597) and the recommended daily intake (365) was 232 mg. Values obtained in this study show that the magnesium content of *Cannabis sativa* inflorescences is almost three times higher than the recommended daily intake. Magnesium is an essential cofactor for the majority of enzyme systems that control a wide range of biochemical activities in the body, including for blood glucose management, muscle and neuron function, protein synthesis and blood pressure regulation (Uusiku et al. 2010). Study findings suggest that the consumption of *Cannabis sativa* and value-added products harvested from the open space environment and chemical fertiliser could assist in curbing conditions such as diabetes, hypertension, and heart-related diseases (Tuckeldoe et al. 2023).

For phosphorus content, results ranged from 198 to 609 mg/100 g DW. Loam soil (control) under the shade net environment reduced phosphorus content from 609 to 198 mg/100 g DW, whereas the open space environment combined with chemical fertiliser increased it from 198 to 609 mg/100 g DW. The variation between the highest phosphorus (609) and recommended daily intake (975) was 366 mg, meaning that *Cannabis sativa* value-added products could contribute about 62% of the phosphorus required by people daily. Phosphorus is needed mostly for the synthesis of bones and teeth, but it also has a significant impact on how the body utilises fats and carbohydrates (Maluleke et al. 2024). In addition, the body needs it to produce protein for tissue and cell growth, maintenance, and repair (Akbari et al. 2022). Therefore, consumption of *Cannabis sativa* and its value-added products grown in an open space environment with chemical fertiliser could potentially assist in preventing conditions such as loss of appetite, bone pain, fatigue, respiratory challenges, and body-weight challenges, which are symptoms linked to low phosphorus in the human diet (Maluleke et al. 2021).

Concerning potassium, results ranged from 349 to 989 mg/100 g DW. The combination of loam soil (control) and the shade net environment reduced potassium content from 989 to 349 mg/100 g DW, whereas the treatment combination of an open space environment and chemical fertiliser increased it from 349 to 989 mg/100 g DW. The variation between the highest potassium content (989) and recommended daily intake (2 850) was 1 861 mg, which implies that *Cannabis sativa* could contribute about 35% of phosphorus required by humans daily. The values obtained from this study indicate that consumption of *Cannabis sativa* and value-added products from an open space environment and chemical fertiliser could assist in curbing conditions such as fatigue, muscle cramps and abnormal heartbeat, which are associated with low potassium in the human diet (Zhang et al. 2021; Tuckeldoe et al. 2023).

**Fig. 6:**
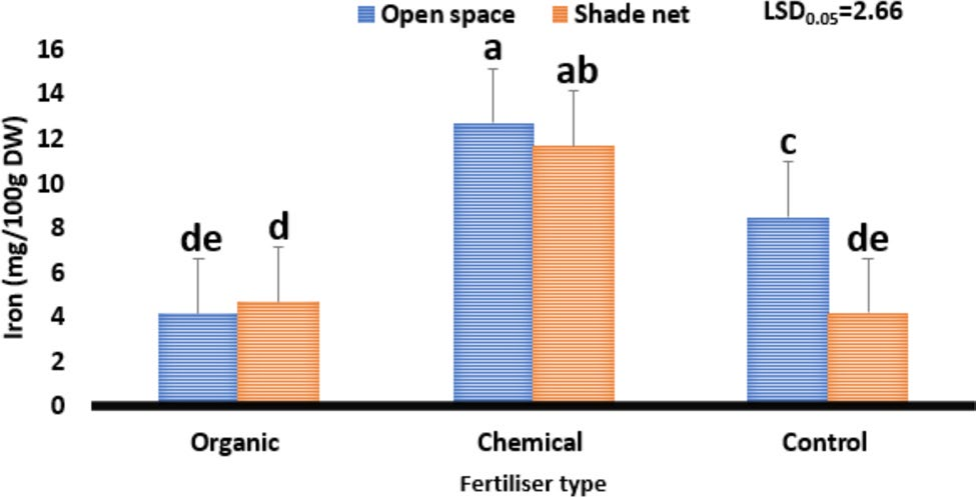
The effect of varying fertiliser types on the iron content (mg/100 DW) of *Cannabis sativa* grown under different environments. The least significant difference, or LSD0.05, is used. Means with different letters (a, b, c) are significantly different at *P* ≤ 0.05

Regarding iron content, results ranged from 4.17 to 11.7 mg/100 g DW as shown in Fig. 6. Moreover, the treatment combination of organic fertiliser and the open space environment reduced iron content from 11.7 to 4.17, whereas the shade net environment combined with chemical fertiliser increased it from 4.2 to 11.7 mg/100 g DW. The variation between the highest iron content (4.17) and the recommended daily intake (17.5) was 13.3 mg. This means that *Cannabis sativa* could contribute about 23% of the iron required by humans daily. Even though the iron content value obtained from the study is low, the consumption of *Cannabis sativa* and its value-added products from the shade net environment and chemical fertiliser could assist in curbing conditions such as constant fatigue, skin-related challenges, chest pain, abnormal heartbeat and inflammation, which are linked to low iron content in the human diet (Borochov-Neori et al. 2008; Uusiku et al. 2010; Achaglinkame et al. 2019).

## Conclusion

The goal of this study was to determine the impact of combined factors (growing environments and fertiliser types) on the physiology, yield and biochemical constituents of *Cannabis sativa* L. The study findings reveal that chemical/inorganic fertiliser was the main contributor in terms of physiological (chlorophyll and stomatal conductance) performance of the plant, regardless of the growing environment. For yield component, such as harvest index and water content, the treatment combination of chemical/inorganic fertiliser and the shade net environment proved to be the best combination when compared to other treatments. Biochemical constituents such as vitamin C, phenols and flavonoids were superior in the treatment combination of the shade net environment and chemical/inorganic fertiliser, whereas macro-nutrients (magnesium, phosphorus, potassium) were higher in the treatment combination of an open space environment and chemical/inorganic fertiliser. The micro-nutrient iron was superior under the treatment combination of a shade net environment and chemical fertiliser. This study suggests that growers should be mindful of plant growth factors such as the environment and fertiliser types, which aid in the production of high-quality plant material for both the medical and food industry through creating value-added products. Moreover, the production of sufficient quality plant material of *Cannabis sativa* creates stability and sustainability in the commercialisation of *Cannabis sativa*, subsequently achieving Sustainable Development Goals SDG 1 (no poverty) and 2 (zero hunger).

## Data Availability

The data generated for this study are available from the corresponding author on formal request.

## Declarations

The authors declare that the manuscript is original.
